# Chapparvoviruses occur in at least three vertebrate classes and have a broad biogeographic distribution

**DOI:** 10.1099/jgv.0.000671

**Published:** 2017-03-13

**Authors:** William Marciel de Souza, Marilia Farignoli Romeiro, Marcílio Jorge Fumagalli, Sejal Modha, Jansen de Araujo, Luzia Helena Queiroz, Edison Luiz Durigon, Luiz Tadeu Moraes Figueiredo, Pablo Ramiro Murcia, Robert James Gifford

**Affiliations:** ^1^​ MRC-University of Glasgow Centre for Virus Research, Glasgow, UK; ^2^​ Virology Research Center, School of Medicine of Ribeirão Preto, University of São Paulo, Ribeirão Preto, Brazil; ^3^​ Institute of Biomedical Sciences, University of São Paulo, São Paulo, Brazil; ^4^​ Faculty of Veterinary Medicine, São Paulo State University, Araçatuba, Brazil

**Keywords:** parvovirus, evolution, virus discovery, metagenomics, endogenous virus

## Abstract

Chapparvoviruses are a highly divergent group of parvoviruses (family *Parvoviridae*) that have recently been identified via metagenomic sampling of animal faeces. Here, we report the sequences of six novel chapparvoviruses identified through both metagenomic sampling of bat tissues and *in silico* screening of published vertebrate genome assemblies. The novel chapparvoviruses share several distinctive genomic features and group together as a robustly supported monophyletic clade in phylogenetic trees. Our data indicate that chapparvoviruses have a broad host range in vertebrates and a global distribution.

## Full-Text

Parvoviruses are small, non-enveloped viruses that have ssDNA genomes ~5 kb in length. They encode two gene cassettes: a non-structural replicase gene (NS) that encodes the enzymes required for replication and a capsid (VP) gene encoding structural proteins. Two parvovirus subfamilies are recognized: *Densovirinae*, which contains viruses that infect invertebrate hosts, and *Parvovirinae*, which contains viruses that infect vertebrate hosts. A total of eight genera have been recognized within the subfamily *Parvovirinae* [1–3]. Here, we report the identification via sequencing of six new members of the recently proposed genus *Chapparvovirus*. We use these data to examine the genome structures and evolutionary relationships of these novel viruses.

All previously described chapparvoviruses have been detected by metagenomic sequencing. The prototypic member of the proposed genus, *Eidolon helvum parvovirus 2* (EhPV-2), was identified in throat swabs taken from the fruit bat *Eidolon helvum* [4]. Three additional chapparvovirus sequences have been identified via metagenomic sequencing of turkey faeces [3], rat faeces [5] and rectal swabs of pigs [6]. It is currently not known whether these viruses are associated with disease.

**Fig. 1.**
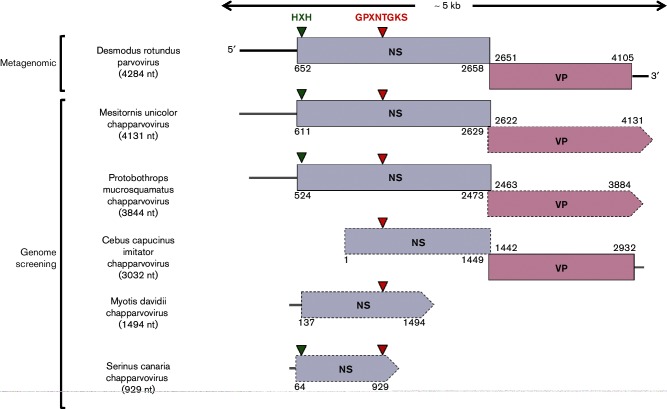
Genome structures of novel parvovirus reported here. The length of the determined nucleotide sequences of the viral sequences is shown in parentheses. Solid-lined boxes and dashed-lined arrows indicate complete or truncated sequence of ORFs, respectively. Truncated termini of ORFs are indicated by an arrow-shaped edge. ORFs were inferred by manual comparison of putative peptide sequences to those of closely related exogenous parvoviruses. Green and red arrowheads on NS1 indicate the position of conserved amino acid motifs of parvoviruses.

The first of six novel chapparvoviruses identified in our study was recovered via metagenomic sequencing of tissue samples derived from common vampire bats (*Desmodus rotundus*). Kidney samples were obtained from eight *D. rotundus* individuals captured in a rural area of Araçatuba city, São Paulo State, Brazil, in June 2010. Pooled samples were used to generate cDNAs and prepared for high-throughput sequencing using TruSeq Universal Adapter (Illumina) protocols (rapid module) and standard multiplex adaptors. A paired-end, 150-base-read protocol in rapid module was used for sequencing on an Illumina HiSeq 2500 instrument as recommended by the manufacturer's protocol. A total of 7 133 306 paired-end reads were generated with 78.12 % of bases ≥Q30 (with a base call accuracy of 99.9 %). Assembly of Illumina reads using metaViC [7] led to recovery of a sequence spanning a near-complete parvovirus genome (Fig. 1). Phylogenetic and genomic analysis established that this sequence represented a virus closely related to EhPV-2, which we refer to as Desmodus rotundus parvovirus (DrPV-1). The DrPV-1 genome is 4284 nt in size and has a typical parvovirus genome organization (Fig. 1).

An additional five chapparvovirus sequences were identified by *in silico* screening of whole-genome shotgun (WGS) sequence assemblies in various databases. The database-integrated genome screening tool [8] was used to screen WGS data of 281 vertebrate species (Table S1, available in the online Supplementary Material) for sequences homologous to parvoviral proteins and to tentatively classify these sequences into genera. This screen identified all previously identified parvovirus endogenous viral elements (EVEs) – all of which group closely with the *Dependoparvovirus* and *Protoparvovirus* genera – and a small number of novel ones. Most of the novel sequences disclosed homology to dependoparvoviruses, protoparvoviruses or amdoparvoviruses (Table S2), but unexpectedly, five disclosed homology to chapparvoviruses. Each of these was identified in a distinct species genome assembly. Two were identified in WGS assemblies of mammalian species, including a bat (*Myotis davidii*) and a New World primate, the white-headed capuchin (*Cebus imitator*). Further, chapparvovirus-related sequences were obtained from WGS assemblies of a reptile, the brown spotted pit viper (*Protobothrops mucrosquamatus*), and two avian species, the Atlantic canary (*Serinus canaria*) and the brown mesite (*Mesitornis unicolor*).

**Fig. 2.**
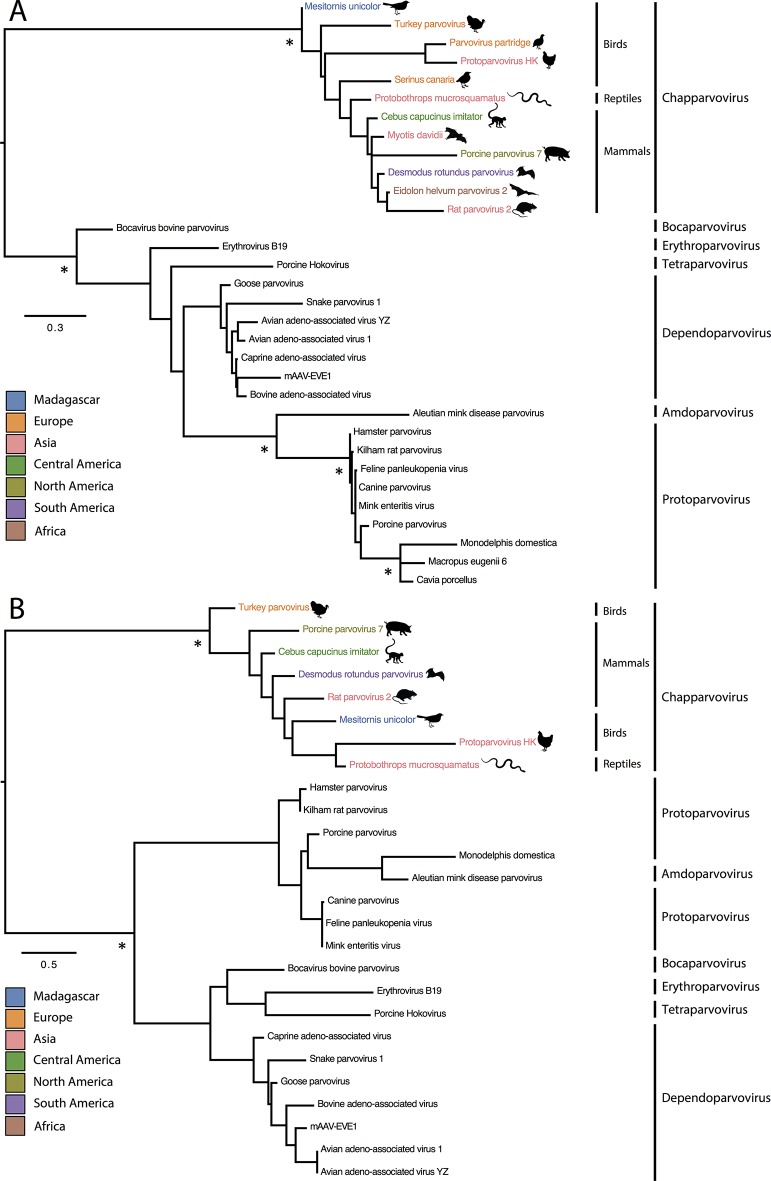
ML phylogenies showing the evolutionary relationships of chapparvoviruses. (a) Phylogeny constructed using an alignment of NS1 proteins and based on RtREV+G protein substitution model. (b) Phylogeny constructed using an alignment of VP proteins and based on LG+I+G protein substitution model. Phylogenies were constructed using RaxML [18], and the protein substitution models were selected by ProtTest [19]. Phylogenies are midpoint rooted for clarity of presentation. The scale bar indicates evolutionary distance in numbers of substitutions per amino acid site. Colours on chapparvovirus branches indicate the geographic associations of isolates (see Table 1), as indicated in the legend. Asterisks indicate nodes with ML bootstrap support levels >75 % based on 1000 bootstrap replicates.

Sequences derived from parvoviruses are known to occur as EVEs in a wide range of animal genomes [9–12]. These sequences are thought to represent the remnants of ancient viruses that became fully or partially integrated into the germline of their hosts through non-homologous recombination events. However, all of the chapparvovirus-related sequences identified in WGS assemblies occurred within relatively short contigs, and since none contained any sequence that we could unambiguously identify as genomic, we could not definitively determine whether they represented integrated sequences (EVEs) or were sequences of exogenous viral DNAs that were present in the original DNA sample from which WGS genome data were generated. Notably, however, none of the sequences showed any evidence of a lengthy residence in the host germline (e.g. stop codons, frameshifting mutations in viral ORFs, transposable element insertions). In addition, all previously described parvovirus EVEs group within or close to the relatively closely related *Dependoparvovirus, Protoparvovirus* and *Amdoparvovirus* genera (Fig. 2). The *Chapparvovirus* genus is only distantly related to these two genera and is separated from them in phylogenies by three other genera (*Bocaparvovirus*, *Tetraparvovirus* and *Erythroparvovirus*) that do not appear to have generated any EVEs (based on current information). Together, these data suggest that the chapparvovirus sequences we identified in WGS assemblies are likely to be infectious viruses present in the DNA samples used for shotgun sequencing, rather than EVEs.

The amino acid sequence identities of novel chapparvovirus sequences shared to those previously published in GenBank were 34–75 % in replicase and 41–55 % in capsid. In contigs that included a complete replicase ORF, the predicted gene product was ~650–672 amino acids in length. Conserved amino acid motifs ‘HVH’ and ‘GPXNTGKS’, the putative endonuclease metal coordination motif ‘HIH’ and the helicase motif ‘GPASTGKS’ were all present (Fig. 1) [13, 14].

As shown in Fig. 1, all six sequences spanned at least part of the replicase gene, including a region that is relatively well conserved across all viruses in the subfamily *Parvovirinae.* We constructed a multiple sequence alignment spanning 113 residues within this region and containing representative *Parvovirinae* reference sequences in addition to novel sequences. A combination of automated procedures (mafft, muscle, blast) and manual adjustment were used to create the final multiple sequence alignment [15–17], which was then translated and used to infer phylogenetic relationships, using maximum likelihood (ML) as implemented in RAxML, and an evolutionary model selected using ProtTest [18, 19]. As shown in Fig. 2(a), all six novel parvovirus sequences robustly group with previously characterized chapparvoviruses in bootstrapped ML trees. Furthermore, within the chapparvovirus group, sequences were observed to cluster into avian, reptilian and mammalian lineages.

Complete capsid ORFs are present in two previously obtained chapparvovirus genome sequences (rat parvovirus and porcine parvovirus 7) and two that were obtained in our study (DrPV-1 and Cebus capucinus parvovirus). Where complete capsid ORFs are present, they are significantly shorter than found in other members of the subfamily *Parvovirinae* (i.e. ~500 amino acids as compared to ~700). Also, the predicted capsid proteins of newly characterized chapparvoviruses contained phospholipase A2 motifs in their N-terminal regions. These motifs, which are reportedly involved in intracellular trafficking and/or escape from endosomes, are found in many, but not all, members of the *Parvoviridae* [20, 21]. Notably, they have been reported to be absent from previously reported chapparvovirus sequences [5, 6]. Phylogenetic relationships between capsid sequences were inferred using the methodology described above for replicase, and those mirrored those obtained for replicase (Fig. 2b).

We noted that the replicase and capsid genes of chapparvoviruses often overlap slightly (~8–11 nucleotides; see Table S3), a trait that has only been observed in one other genus (*Erythroparvovirus*) within the *Parvovirinae*. The relatively small size of the chapparvovirus capsid protein, combined with the presence of overlap between the capsid and replicase genes, suggests a selection pressure for smaller genome size in these viruses. If we assume that the capsid gene found in these viruses shares a common origin with those found in other *Parvovirinae* genera, then it appears that this genus has evolved a smaller overall genome size, reducing the size of the capsid gene, while the replicase gene has remained approximately unchanged. Interestingly, this goes against the well-established hypothesis that virus genome size is physically limited by length constraints on genes encoding icosahedral capsids [22–24]. However, since we could not identify any regions of unambiguous homology between the chapparvovirus capsid proteins and those found in other *Parvovirinae* genera, an alternative scenario can also be considered wherein the shorter chapparvovirus capsid gene has a separate evolutionary origin to the one found in the other genera.

The clustering of chapparvovirus sequences into host-class-specific sub-lineages (see Fig. 2) is consistent with their being derived from viruses that have been evolutionarily associated with their different hosts. We collected information on the location and context of sampling for samples that were used to generate the metagenomic and WGS sequence datasets, and mapped the biogeographic associations of samples onto the replicase phylogeny (Table 1, Fig. 2). These data show that chapparvoviruses have an extensive geographic distribution and likely have a worldwide distribution in many different hosts. In future studies, we expect that these viruses will be found in many other hosts – perhaps without causing disease in most cases.

**Table 1.** Sample information names, sources, sample, locality and environment of viruses reported in this study Accession numbers: DrPV-1 (KX907333), Cebus capucinus imitator chapparvovirus (LVWQ01135885), Mesitornis unicolor chapparvovirus (JJRI01094129), Protobothrops mucrosquamatus chapparvovirus (BCNE02131058), Myotis davidii chapparvovirus (ALWT01091740), Serinus canaria chapparvovirus (CAVT010188449).

Virus	Source	Sample	Location	Date	Environment
DrPV-1	*D. rotundus*	Pool of kidney	Araçatuba city, São Paulo, Brazil	23 June 2010	Native
Cebus capucinus imitator chapparvovirus	*C. capucinus imitator* (adult male)	Missing	Costa Rica	Missing	Missing – killed by a vehicle
Mesitornis unicolor chapparvovirus	*Mesitornis unicolor* (female)	Missing	Madagascar	Missing	Missing
Protobothrops mucrosquamatus chapparvovirus	*P. mucrosquamatus*	Missing	Okinawa, Japan	2014	Missing
Myotis davidii chapparvovirus	*Myotis davidii*	Spleen, kidney and small intestine	Taiyi Cave, Xianning, China	21 August 2011	Native
Serinus canaria chapparvovirus	*S. canaria*	Missing	Missing	Missing	Missing

## Supplementary Data

456 Supplementary File 1
