# Supplementary material for: Chapparvoviruses occur in at least three vertebrate classes and have a broad biogeographic distribution
Source: J Gen Virol. 2017 Mar 13;98(2):225–9. doi: 10.1099/jgv.0.000671 (PMC5646239; doi:10.1099/jgv.0.000671)
Supplement: Supplementary File 1 [file jgv-98-225-s001.pdf]

Table S1. Vertebrate genome assemblies screened

Species	Class	Genome assembly version
Amphilophus citrinellus	Actinopterygii	Midas_v5
Anguilla anguilla	Actinopterygii	v1
Anguilla japonica	Actinopterygii	v1_25_oct_2011
Anguilla rostrata	Actinopterygii	v1
Anoplopoma fimbria	Actinopterygii	AnoFim1.0
Astyanax mexicanus	Actinopterygii	ensembl_79_AstMex102
Austrofundulus limnaeus	Actinopterygii	1
Boleophthalmus pectinirostris	Actinopterygii	1_BP
Clupea harengus	Actinopterygii	ncbi_7950_2015
Cottus rhenanus	Actinopterygii	v1
Cynoglossus semilaevis	Actinopterygii	cse_v1.0
Cyprinodon nevadensis pectoralis	Actinopterygii	v1
Cyprinodon variegatus	Actinopterygii	C_variegatus-1.0
Cyprinus carpio	Actinopterygii	v1_2015
Danio rerio	Actinopterygii	ensembl_79_Zv9
Dicentrarchus labrax	Actinopterygii	seabass_V1.0
Esox lucius	Actinopterygii	ASM72191v2
Fundulus heteroclitus	Actinopterygii	3.0.2
Gadus morhua	Actinopterygii	goldenpath_gadMor1_2010_05
Gasterosteus aculeatus	Actinopterygii	BROADS1
Haplochromis burtoni	Actinopterygii	1
Ictalurus punctatus	Actinopterygii	IpCoco_1.2
Kryptolebias marmoratus	Actinopterygii	v1
Labeotropheus fuelleborni	Actinopterygii	v1
Larimichthys crocea	Actinopterygii	ASM74293v1
Lates calcarifer	Actinopterygii	ASM164080v1
Lepisosteus oculatus	Actinopterygii	LepOcu1
Leuciscus waleckii	Actinopterygii	Amur_ide_v1
Leucoraja erinacea	Actinopterygii	LER_WGS_1
Maylandia zebra	Actinopterygii	UMD1
Mchenga conophoros	Actinopterygii	ASM15085v1
Miichthys miiuy	Actinopterygii	ASM159371v1
Mola mola	Actinopterygii	ASM169857v1
Neolamprologus brichardi	Actinopterygii	NeoBri1.0
Nothobranchius furzeri	Actinopterygii	Nfu_20140520
Notothenia coriiceps	Actinopterygii	ncbi_8208_2014
Oreochromis niloticus	Actinopterygii	goldenpath_oreNil2_2011_01

Oryzias latipes	Actinopterygii	medaka1
Pampus argenteus	Actinopterygii	PamArg1.0
Periophthalmodon schlosseri	Actinopterygii	PS
Periophthalmus magnuspinnatus	Actinopterygii	GCA_000787105
Pimephales promelas	Actinopterygii	GCA_000700825.1
Poecilia formosa	Actinopterygii	PoeFor_5.1.2
Poecilia latipinna	Actinopterygii	v1.0
Poecilia mexicana	Actinopterygii	v1.0
Poecilia reticulata	Actinopterygii	v1.0
Pseudopleuronectes yokohamae	Actinopterygii	Pyoko_1.0
Pundamilia nyererei	Actinopterygii	PunNye1.0
Pygocentrus nattereri	Actinopterygii	1.0.2
Rhamphochromis esox	Actinopterygii	ASM15093v1
Salmo salar	Actinopterygii	ICASG_v2
Scartelaos histophorus	Actinopterygii	SH
Scleropages formosus	Actinopterygii	aro_v2
Sebastes nigrocinctus	Actinopterygii	Snig1.0
Sebastes rubrivinctus	Actinopterygii	SRub1.0
Sinocyclocheilus anshuiensis	Actinopterygii	SAMN03320099
Sinocyclocheilus grahami	Actinopterygii	v1.1
Sinocyclocheilus rhinoceros	Actinopterygii	SAMN03320098_v1
Stegastes partitus	Actinopterygii	1.0.2
Takifugu flavidus	Actinopterygii	v1
Takifugu rubripes	Actinopterygii	FUGU4
Tetraodon nigroviridis	Actinopterygii	TETRAODON8
Thunnus orientalis	Actinopterygii	Ba_1.0
Xiphophorus couchianus	Actinopterygii	4.0.1
Xiphophorus hellerii	Actinopterygii	3.0.1
Xiphophorus maculatus	Actinopterygii	ncbi_100_2013
Lethenteron camtschaticum	Agnatha	LetJap1.0
Petromyzon marinus	Agnatha	Pmarinus_7.0
Ambystoma mexicanum	Amphibia	sung-2015
Cynops pyrrhogaster	Amphibia	sung-2016
Hynobius chinensis	Amphibia	sung-2015
Nanorana parkeri	Amphibia	GCA_000935625.1
Notophthalmus viridescens	Amphibia	sung-2015
Triturus carnifex	Amphibia	sung_2016
Xenopus tropicalis	Amphibia	v7_2015-06-19
Acanthisitta chloris	Aves	bgi_2011-10-28
Amazona aestiva	Aves	ASM142067v1
Amazona vittata	Aves	AV1

Anas platyrhynchos	Aves	ensembl_79_BGI_duck_1.0
Anas platyrhynchos	Aves	bgi_2011-10-28
Anser cygnoides	Aves	PRJNA183603
Antrostomus carolinensis	Aves	bgi_2011-10-28
Apaloderma vittatum	Aves	bgi_2011-10-28
Aptenodytes forsteri	Aves	bgi_2011-10-28
Apteryx australis mantelli	Aves	AptMant0
Aquila chrysaetos canadensis	Aves	ncbi_100
Ara macao	Aves	SMACv1.1
Balearica regulorum	Aves	bgi_2011-10-28
Buceros rhinoceros	Aves	bgi_2011-10-28
Calidris pugnax	Aves	ASM143184v1
Calypte anna	Aves	bgi_2011-10-28
Cariama cristata	Aves	bgi_2011-10-28
Cathartes aura	Aves	bgi_2011-10-28
Chaetura pelagica	Aves	bgi_2011-10-28
Charadrius vociferus	Aves	bgi_2011-10-28
Chlamydotis macqueenii	Aves	ASM69519v1
Chlamydotis undulata	Aves	bgi_2011-10-28
Colinus virginianus	Aves	NB1.1
Colius striatus	Aves	bgi_2011-10-28
Columba livia	Aves	bgi_2011-10-28
Corvus brachyrhynchos	Aves	bgi_2011-10-28
Corvus cornix cornix	Aves	932674
Coturnix japonica	Aves	ncbi_2.0
Cuculus canorus	Aves	bgi_2011-10-28
Egretta garzetta	Aves	bgi_2011-10-28
Eurypyga helias	Aves	bgi_2011-10-28
Falco cherrug	Aves	F_cherrug_v1.0
Falco peregrinus	Aves	bgi_2011-10-28
Ficedula albicollis	Aves	FicAlb_1.4
Fulmarus glacialis	Aves	bgi_2011-10-28
Gallus gallus	Aves	ensembl_Galgal4
Gallus gallus	Aves	goldenpath_galGal4
Gavia stellata	Aves	bgi_2011-10-28
Geospiza fortis	Aves	goldenpath_geoFor1
Haliaeetus albicilla	Aves	bgi_2012-3-16
Haliaeetus leucocephalus	Aves	bgi_2012-03-16
Lepidothrix coronata	Aves	1
Leptosomus discolor	Aves	bgi_2011-10-28
Lyrurus tetrix	Aves	tetTet1

Manacus vitellinus	Aves	28/10/2011
Meleagris gallopavo	Aves	goldenpath_melGal1
Melopsittacus undulatus	Aves	goldenpath_melUnd1
Merops nubicus	Aves	bgi_2011-10-28
Mesitornis unicolor	Aves	bgi_2011-10-28
Nestor notabilis	Aves	bgi_2011-10-28
Nipponia nippon	Aves	bgi_2011-10-28
Opisthocomus hoazin	Aves	bgi_2011-10-28
Parus major	Aves	1.0.3
Pelecanus crispus	Aves	bgi_2011-10-28
Phaethon lepturus	Aves	bgi_2011-10-28
Phalacrocorax carbo	Aves	bgi_2011-10-28
Phoenicopterus ruber	Aves	bgi_2011-10-28
Phylloscopus plumbeitarsus	Aves	GWplu1.0
Phylloscopus trochiloides viridanus	Aves	GWvir1.0
Picoides pubescens	Aves	bgi_2011-10-28
Podiceps cristatus	Aves	bgi_2011-10-28
Pseudopodoces humilis	Aves	1
Pterocles gutturalis	Aves	bgi_2011-10-28
Pygoscelis adeliae	Aves	bgi_2011-10-28
Serinus canaria	Aves	ncbi_100
Struthio camelus	Aves	bgi_2011-10-28
Sturnus vulgaris	Aves	GCA_001447265.1
Taeniopygia guttata	Aves	goldenpath_taeGut2
Tauraco erythrolophus	Aves	bgi_2011-10-28
Tinamus guttatus	Aves	bgi_2011-10-28
Tyto alba	Aves	bgi_2011-10-28
Zonotrichia albicollis	Aves	1.0.1
Zosterops lateralis	Aves	ASM128173v1
Callorhinchus milii	Chondrichthyes	goldenpath_calMil1_2013-12
Rhincodon typus	Chondrichthyes	Rhincodon_v1
Alligator mississippiensis	Crocodilia	goldenpath_allMis1_2012
Alligator sinensis	Crocodilia	ASM45574v1
Crocodylus porosus	Crocodilia	crocgenomes_sub2
Gavialis gangeticus	Crocodilia	ggan_v0.2
Acinonyx jubatus	Mammalia	aciJub1
Ailuropoda melanoleuca	Mammalia	ensembl_79_ailMel1
Ailuropoda melanoleuca	Mammalia	goldenpath_ailMel1
Aotus nancymaae	Mammalia	ncbi_1.0_April_2015
Apodemus sylvaticus	Mammalia	GCA_001305905.1
Balaenoptera acutorostrata	Mammalia	goldenpath_balAcu1

Balaenoptera bonaerensis	Mammalia	ASM97880v1
Bison bison	Mammalia	ncbi_UMD1_2015-05-14
Bos grunniens	Mammalia	bbu_UMD_2015-05-14
Bos mutus	Mammalia	v2.0
Bos taurus	Mammalia	ensembl_79_UMD3.1
Bos taurus	Mammalia	goldenpath_bosTau7
Bubalus bubalis	Mammalia	ncbi_100_2015-05-14
Callithrix jacchus	Mammalia	ensembl_79_C_jacchus3.2.1
Callithrix jacchus	Mammalia	goldenpath_calJac3
Camelus bactrianus	Mammalia	MBC_1.0
Camelus dromedarius	Mammalia	ncbi_GCF_000767585_2015-05-14
Camelus ferus	Mammalia	bacsac_1.0_april_2013
Canis familiaris	Mammalia	ensembl_79_CanFam3.1
Canis familiaris	Mammalia	goldenpath_canFam3
Capra aegagrus	Mammalia	CapAeg_1.0
Capra hircus	Mammalia	iggc_1.0_april_2013
Capreolus capreolus	Mammalia	kmer631
Cavia aperea	Mammalia	CavAp1.0
Cavia porcellus	Mammalia	ensembl_79_cavPor3
Cavia porcellus	Mammalia	CHO-K1-v1
Cavia porcellus	Mammalia	goldenpath_cavPor3
Cebus imitator	Mammalia	Cebus_imitator-1.0
Ceratotherium simum	Mammalia	goldenpath_cerSim1
Cercocebus atys	Mammalia	ncbi_100_2015
Chinchilla lanigera	Mammalia	broad_v0_2012
Chlorocebus sabaeus	Mammalia	ensembl_79_ChlSab1.1
Choloepus hoffmanni	Mammalia	ensembl_79_choHof1
Choloepus hoffmanni	Mammalia	goldenpath_choHof1
Chrysochloris asiatica	Mammalia	broad_ChrAsi1.0_2012
Colobus angolensis palliatus	Mammalia	ncbi_100_2015
Condylura cristata	Mammalia	broad_ConCri1.0
Cricetulus griseus	Mammalia	goldenpath_criGri1
Dasypus novemcinctus	Mammalia	ensembl_79_Dasnov3.0
Dasypus novemcinctus	Mammalia	goldenpath_dasNov3
Daubentonia madagascariensis	Mammalia	broad_1.0_oct_2012
Dipodomys ordii	Mammalia	ensembl_79_dipOrd1
Echinops telfairi	Mammalia	ensembl_79_TENREC
Echinops telfairi	Mammalia	goldenpath_echTel2
Eidolon helvum	Mammalia	GCA_000465285.1
Elephantulus edwardii	Mammalia	broad_1.0_april_2013
Ellobius lutescens	Mammalia	ASM168507v1

Ellobius talpinus	Mammalia	ETalpinus_0.1
Eptesicus fuscus	Mammalia	broad_1.0_april_2013
Equus africanus asinus	Mammalia	willy_denovo
Equus caballus Icelandic	Mammalia	Henan_EquCab2_17Aug
Equus caballus arabian	Mammalia	Henan_EquCab2_Jun15
Equus caballus connemara	Mammalia	Henan_EquCab2_17Aug
Equus caballus fjrod	Mammalia	Henan_EquCab2_17Aug
Equus caballus mongolian	Mammalia	GCA_000696655
Equus caballus mongolian	Mammalia	GCA_000696655_NCBI
Equus caballus standardbred	Mammalia	Henan_EquCab2_17Aug
Equus caballus	Mammalia	ensembl_79_EquCab2
Equus caballus	Mammalia	goldenpath_equCab2_Dec13
Equus ferus przewalskii	Mammalia	ncbi_9798_15Dec2014
Equus ferus thistlecreek	Mammalia	Henan_EquCab2_Jun30
Erinaceus europaeus	Mammalia	ensembl_79_HEDGEHOG
Erinaceus europaeus	Mammalia	goldenpath_eriEur2
Eulemur flavifrons	Mammalia	Eflavifronsk33QCA
Eulemur macaco	Mammalia	Emacaco_refEf
Felis catus	Mammalia	ensembl_79_Felis_catus_6.2
Felis catus	Mammalia	goldenpath_felCat5
Fukomys damarensis	Mammalia	ncbi_1.0_2014
Galeopterus variegatus	Mammalia	G-variegatus_3.0.2_apr_2015
Giraffa camelopardalis tippelskirchi	Mammalia	ASM165123v1
Gorilla gorilla	Mammalia	ensembl_79_gorGor3.1
Gorilla gorilla	Mammalia	goldenpath_gorGor3
Heterocephalus glaber	Mammalia	goldenpath_hetGla2
Homo sapiens	Mammalia	ensembl_79_GRCh38
Homo sapiens	Mammalia	goldenpath_hg19_2009
Homo sapiens	Mammalia	goldenpath_hg38
Homo sapiens	Mammalia	goldenpath_hg38_chromosome
Ictidomys tridecemlineatus	Mammalia	ensembl_79_spetri2
Jaculus jaculus	Mammalia	broad_JacJac1.0
Lemur catta	Mammalia	26/06/2015
Leptonychotes weddellii	Mammalia	broad_1.1_april_2013
Lipotes vexillifer	Mammalia	ncbi_100_2014-04-27
Loxodonta africana	Mammalia	ensembl_79_loxAfr3
Loxodonta africana	Mammalia	goldenpath_loxAfr3
Macaca fascicularis	Mammalia	5
Macaca mulatta	Mammalia	ensembl_79_MMUL_1
Macaca mulatta	Mammalia	goldenpath_rheMac3
Macaca nemestrina	Mammalia	Mnem_1.0

Macropus eugenii	Mammalia	ensembl_79_Meug_1.0
Macropus eugenii	Mammalia	goldenpath_macEug2
Mandrillus leucophaeus	Mammalia	Mleu.le_1.0
Manis javanica	Mammalia	ManJav1.0
Manis pentadactyla	Mammalia	GCA_000738955.1
Marmota marmota marmota	Mammalia	marMar2.1
Megaderma lyra	Mammalia	ncbi_AWHB00000000.1_2015-05
Mesocricetus auratus	Mammalia	broad_1.1_april_2013
Microcebus murinus	Mammalia	ensembl_79_micMur1
Microcebus murinus	Mammalia	goldenpath_micMur1
Microtus ochrogaster	Mammalia	broad_1.0_april_2013
Miniopterus natalensis	Mammalia	Mnat.v1
Monodelphis domestica	Mammalia	ensembl_79_BROADO5
Monodelphis domestica	Mammalia	goldenpath_monDom5
Mus caroli	Mammalia	REL-1509_EiJ
Mus castaneus	Mammalia	REL-1509-EiJ
Mus musculus	Mammalia	ensembl_79_GRCm38
Mus musculus	Mammalia	goldenpath_mm10
Mus musculus	Mammalia	129S1_SvImJ
Mus musculus	Mammalia	A_J
Mus musculus	Mammalia	BALB_cJ
Mus musculus	Mammalia	C3H_HeJ
Mus musculus	Mammalia	CBA_J
Mus musculus	Mammalia	DBA_2J
Mus musculus	Mammalia	FVB_NJ
Mus musculus	Mammalia	LP_J
Mus musculus	Mammalia	NOD_ShiLtJ
Mus musculus	Mammalia	NZO_HlLtJ
Mus pahari	Mammalia	REL-1509-EiJ
Mus spretus	Mammalia	SPRET_EiJ_v1
Mustela putorius furo	Mammalia	ensembl_79_MusPutFur1.0
Mustela putorius furo	Mammalia	goldenpath_musFur1
Myodes glareolus	Mammalia	ASM130578v1
Myotis brandtii	Mammalia	ncbi_100_2013
Myotis davidii	Mammalia	bgi_1.1_april_2013
Myotis lucifugus	Mammalia	ensembl_79_Myoluc2.0
Myotis lucifugus	Mammalia	goldenpath_myoLuc2
Nannospalax galili	Mammalia	S.galili_v1.0
Nasalis larvatus	Mammalia	goldepath_nasLar1_2015-12
Neotoma lepida	Mammalia	ASM167557v1
Nomascus leucogenys	Mammalia	ensembl_79_Nleu1.0

Ochotona princeps	Mammalia	ensembl_79_pika
Ochotona princeps	Mammalia	goldenpath_ochPri3
Octodon degus	Mammalia	broad_1.0_july_2012
Odobenus rosmarus	Mammalia	marinemamm_1.0_april_2013
Odocoileus virginianus	Mammalia	RRL-RSL_1.0
Okapia johnstoni	Mammalia	ASM166083v1
Orcinus orca	Mammalia	ncbi_ANOL02_jan_2013
Ornithorhynchus anatinus	Mammalia	ensembl_79_OANA5
Ornithorhynchus anatinus	Mammalia	goldenpath_ornAna1_2007-03
Orycteropus afer	Mammalia	broad_v0_2012
Oryctolagus cuniculus	Mammalia	ensembl_79_OryCun2.0
Oryctolagus cuniculus	Mammalia	goldenpath_oryCun2
Otolemur garnettii	Mammalia	ensembl_79_OtoGar3
Otolemur garnettii	Mammalia	goldenpath_otoGar3
Ovis aires	Mammalia	ensembl_79_Oar_v3.1
Ovis aires	Mammalia	goldenpath_oviAri3
Pan troglodytes	Mammalia	ensembl_79_CHIMP2.1.4
Pan troglodytes	Mammalia	goldenpath_panTro4
Panthera tigris altaica	Mammalia	ncbi_100_2014-03
Pantholops hodgsonii	Mammalia	PHO1.0
Papio anubis	Mammalia	ensembl_79_PapAnu2.0
Papio anubis	Mammalia	goldenpath_papAnu2
Peromyscus maniculatus bairdii	Mammalia	1
Phodopus sungorus	Mammalia	Psun0.5
Physeter catodon	Mammalia	2.0.2
Pongo abelii	Mammalia	ensembl_79_PPYG2
Pongo abelii	Mammalia	goldenpath_ponAbe2
Procavia capensis	Mammalia	ensembl_79_proCap1
Procavia capensis	Mammalia	goldenpath_proCap1
Propithecus coquereli	Mammalia	Pcoq_1.0
Pteronotus parnellii	Mammalia	ncbi_AWGZ00000000.1_2015-05
Pteropus alecto	Mammalia	broad_1.0_april_2012
Pteropus vampyrus	Mammalia	ensembl_79_pteVam1
Pteropus vampyrus	Mammalia	goldenpath_pteVam1
Rattus norvegicus	Mammalia	ensembl_79_Rnor_5.0
Rattus norvegicus	Mammalia	goldenpath_rn6
Rhinolophus ferrumequinum	Mammalia	ncbi_AWHA00000000.1_2015-05
Rhinopithecus bieti	Mammalia	ASM169854v1
Rhinopithecus roxellana	Mammalia	ncbi_100_2014-11-18
Rousettus aegyptiacus	Mammalia	2
Saimiri boliviensis	Mammalia	goldenpath_saiBol1

Sarcophilus harrisii	Mammalia	ensembl_79_DEVIL7.0
Sarcophilus harrisii	Mammalia	goldenpath_sarHar1
Sorex araneus	Mammalia	ensembl_79_COMMON_SHREW1
Sorex araneus	Mammalia	goldenpath_sorAra2
Sus scrofa	Mammalia	ensembl_79_Sscrofa10.2
Sus scrofa	Mammalia	goldenpath_susScr3
Tarsius syrichta	Mammalia	ensembl_tarSyr1
Tarsius syrichta	Mammalia	goldenpath_tarSyr1
Trichechus manatus	Mammalia	goldenpath_triMan1
Tupaia belangeri	Mammalia	ensembl_79_TREESHREW
Tupaia belangeri	Mammalia	goldenpath_tupBel1
Tupaia chinensis	Mammalia	TupChi_1.0
Tursiops truncatus	Mammalia	ensembl_79_turTru1
Tursiops truncatus	Mammalia	goldenpath_turTru2
Ursus maritimus	Mammalia	GAJD01_1.0_june_2013
Vicugna pacos	Mammalia	ensembl_79_vicPac1
Vicugna pacos	Mammalia	goldenpath_vicPac2
Latimeria chalumnae	Sarcopterygii	LatCha1
Anolis carolinensis	Squamata	ensembl_79_AnoCar2.0
Anolis carolinensis	Squamata	goldenPath_anoCar2
Apalone spinifera	Squamata	Apla_1.0
Chelonia mydas	Squamata	CheMyd_1.0
Chrysemys picta	Squamata	goldenpath_chrPic1_2011
Crotalus horridus	Squamata	v1
Crotalus mitchellii pyrrhus	Squamata	CrotMitch1.0
Gekko japonicus	Squamata	V1.1
Malaclemys terrapin terrapin	Squamata	terp_v2_2
Ophiophagus hannah	Squamata	OphHan1.0
Pantherophis guttatus	Squamata	PanGut1.0
Pelodiscus sinensis	Squamata	PelSin_1.0
Protobothrops mucrosquamatus	Squamata	P.Mucros_1.0
Python bivittatus	Squamata	5.0.2
Thamnophis sirtalis	Squamata	6
Vipera berus	Squamata	Vber.be_1.0

Table S2. Sequences disclosing similarity to parvoviruses in whole genome sequence assemblies.

Closest matching exogenous virus sequence in reference library*	Gene match	Genome build	Scaffold	Start	End	Length	Bit score
Aleutian-mink-disease-virus	unknown-protein	C_lanigera_v0.assembly.fasta_24.fa	scaffold00132	460508	460846	339	110
Aleutian-mink-disease-virus	unknown-protein	cavPor3.fa	scaffold_27	12841776	12842051	276	67.4
Aleutian-mink-disease-virus	unknown-protein	proCap1.fa	scaffold_34481	3628	3945	318	78.2
Aleutian-mink-disease-virus	unknown-protein	macEug2.fa	GL106615	53397	53711	315	63.2
Aleutian-mink-disease-virus	unknown-protein	macEug2.fa	GL110448	5021	5251	231	73.6
Aleutian-mink-disease-virus	unknown-protein	macEug2.fa	GL161406	13754	14191	438	73.9
Aleutian-mink-disease-virus	unknown-protein	macEug2.fa	GL106615	52974	53165	192	63.9
Aleutian-mink-disease-virus	unknown-protein	macEug2.fa	GL142423	29306	29641	336	98.6
Aleutian-mink-disease-virus	unknown-protein	macEug2.fa	GL152084	13211	13453	243	68.6
Aleutian-mink-disease-virus	unknown-protein	chr6.fa	chr6	113564550	113564885	336	82
Aleutian-mink-disease-virus	unknown-protein	103944_ref_P.Mucros_1.0_chrUn.fa	gi|1002152152|ref|NW_015386759.1|	97680	98108	429	124
Aleutian-mink-disease-virus	unknown-protein	103944_ref_P.Mucros_1.0_chrUn.fa	gi|1002152152|ref|NW_015386759.1|	98470	98733	264	69.7
Bufavirus-3	VP	Takifugu_rubripes.FUGU4.dna.nonchromosomal.fa	scaffold_3662	4894	5283	390	64.7
Bufavirus-3	VP	Takifugu_rubripes.FUGU4.dna.nonchromosomal.fa	scaffold_8385	1419	2360	942	113
Mpulungu-bufavirus	NS	Takifugu_rubripes.FUGU4.dna.nonchromosomal.fa	scaffold_3621	3106	3279	174	63.9
Mpulungu-bufavirus	NS	Takifugu_rubripes.FUGU4.dna.nonchromosomal.fa	scaffold_3662	6750	6923	174	63.9
Mpulungu-bufavirus	NS	Takifugu_rubripes.FUGU4.dna.nonchromosomal.fa	scaffold_8385	275	448	174	63.9
Mpulungu-bufavirus	NS	Takifugu_rubripes.FUGU4.dna.nonchromosomal.fa	scaffold_2074	18716	18889	174	62
Bufavirus-3	NS	chr6.fa	chr6	48167990	48168229	240	87.8
Bufavirus-3	NS	macEug2.fa	GL089129	21759	22337	579	132
Bufavirus-3	NS	chr6.fa	chr6	113562648	113562800	153	63.9
Bufavirus-3	VP	cavPor3.fa	scaffold_27	12841261	12841524	264	83.2
Bufavirus-3	VP	triMan1.fa_2.fa	JH594625	3921196	3921564	369	122
Bufavirus-3	VP	macEug2.fa	GL051588	33906	34115	210	69.3
Bufavirus-3	VP	macEug2.fa	GL152084	14172	14810	639	112
Bufavirus-3	VP	sarHar1.fa_6.fa	chr6_GL864817_random	149618	149944	327	67
Mpulungu-bufavirus	NS	C_lanigera_v0.assembly.fasta_12.fa	scaffold00033	14639528	14640235	708	308
Mpulungu-bufavirus	NS	C_lanigera_v0.assembly.fasta_24.fa	scaffold00132	461997	462758	762	302
Mpulungu-bufavirus	NS	O_degus_v0.assembly.fasta_18.fa	scaffold00088	366335	367093	759	228
Mpulungu-bufavirus	NS	O_degus_v0.assembly.fasta_33.fa	scaffold00312	1344476	1344757	282	148
Mpulungu-bufavirus	NS	cavPor3.fa	scaffold_188	79736	80047	312	83.6
Mpulungu-bufavirus	NS	cavPor3.fa	scaffold_31	24518452	24518961	510	158
Mpulungu-bufavirus	NS	cavPor3.fa	scaffold_31	24706414	24707040	627	80.9
Mpulungu-bufavirus	NS	cavPor3.fa	scaffold_27	12834368	12834775	408	199
Mpulungu-bufavirus	NS	C_asiatica_v0.assembly.fa	scaffold00010	34329538	34329762	225	95.9
Mpulungu-bufavirus	NS	triMan1.fa_2.fa	JH594625	3923623	3923874	252	116
Mpulungu-bufavirus	NS	macEug2.fa	GL152084	11645	12196	552	276
Mpulungu-bufavirus	NS	macEug2.fa	GL161406	11976	12473	498	177
Mpulungu-bufavirus	NS	macEug2.fa	GL132395	10777	10995	219	89.4
Mpulungu-bufavirus	NS	macEug2.fa	GL106615	51199	51684	486	157
Mpulungu-bufavirus	NS	chr3.fa	chr3	352565740	352566102	363	155
Mpulungu-bufavirus	NS	chr8.fa	chr8	230387503	230387802	300	118
Mpulungu-bufavirus	NS	macEug2.fa	GL156485	10881	11333	453	163
Mpulungu-bufavirus	NS	sarHar1.fa_1.fa	chr1_GL834608_random	1660932	1661699	768	335

Mpulungu-bufavirus	NS	sarHar1.fa_2.fa	chr2_GL841323_random	471422	471721	300	127
Mpulungu-bufavirus	VP	O_degus_v0.assembly.fasta_15.fa	scaffold00062	12296696	12296968	273	83.2
Mpulungu-bufavirus	VP	O_degus_v0.assembly.fasta_21.fa	scaffold00109	3849935	3850645	711	85.9
Mpulungu-bufavirus	VP	O_degus_v0.assembly.fasta_33.fa	scaffold00312	1342515	1343045	531	75.5
Mpulungu-bufavirus	VP	macEug2.fa	GL116559	18533	19402	870	150
Mpulungu-bufavirus	VP	macEug2.fa	GL116559	17991	18272	282	100
Mpulungu-bufavirus	VP	macEug2.fa	GL051588	29008	30390	1383	244
Mpulungu-bufavirus	VP	macEug2.fa	GL142423	28095	29024	930	200
Mpulungu-bufavirus	VP	macEug2.fa	GL161406	14316	14672	357	81.6
Mpulungu-bufavirus	VP	macEug2.fa	GL110448	5581	6075	495	90.5
Mpulungu-bufavirus	VP	macEug2.fa	GL132395	8856	9530	675	81.3
Mpulungu-bufavirus	VP	macEug2.fa	GL106615	53851	54216	366	76.3
Mpulungu-bufavirus	VP	macEug2.fa	GL121440	39155	39517	363	105
Mpulungu-bufavirus	VP	macEug2.fa	GL150710	15685	15870	186	63.5
Mpulungu-bufavirus	VP	macEug2.fa	GL081802	59657	60655	999	190
Mpulungu-bufavirus	VP	chr8.fa	chr8	230395154	230396815	1662	205
Mpulungu-bufavirus	VP	chr3.fa	chr3	352564186	352564737	552	103
Mpulungu-bufavirus	VP	macEug2.fa	GL084993	343	789	447	96.7
Mpulungu-bufavirus	VP	sarHar1.fa_1.fa	chr1_GL834608_random	1660164	1660448	285	109
Adeno-associated-virus-1	VP	Pelecanus_crispus.scaf.noBacterial.fa	scaffold56836	6084	6221	138	67.4
Adeno-associated-virus-2	NS	Pelecanus_crispus.scaf.noBacterial.fa	scaffold56836	7319	7609	291	155
Adeno-associated-virus-2	VP	geoFor1.fa_1.fa	JH739894	4004285	4004548	264	62.4
Adeno-associated-virus-2	VP	Manacus_vitellinus.scaf.noBacterial.fa	scaffold270	152409	152714	306	84.7
Adeno-associated-virus-5	VP	Ophisthocomus_hoazin.scaf.noBacterial.fa	scaffold101	294380	294574	195	70.5
Adeno-associated-virus-8	NS	Pelecanus_crispus.scaf.noBacterial.fa	scaffold1346	88329	88703	375	164
Avian-adeno-associated-virus-strain-DA-1	NS	Egretta_garzetta.scaf.noBacterial.fa	scaffold81	2266872	2267294	423	96.7
Bat-adeno-associated-virus-YNM	VP	taeGut2.fa	chr4	13008834	13009106	273	70.9
Goose-parvovirus	NS	Chlamydotis_undulata.scaf.noBacterial.fa	scaffold13103	415	951	537	127
Goose-parvovirus	NS	Chlamydotis_undulata.scaf.noBacterial.fa	scaffold13103	7620	8009	390	114
Muscovy-duck-parvovirus	VP	Manacus_vitellinus.scaf.noBacterial.fa	scaffold270	153083	153304	222	89.7
Snake-adeno-associated-virus	VP	Acanthisitta_chloris.scaf.noBacterial.fa	scaffold6994	79163	79330	168	63.5
Adeno-associated-virus-5	DNA-binding-trs-helicase	Tetraodon_nigroviridis.TETRAODON8.dna.nonchromosomal.fa	Un_random	94959430	94959906	477	111
Adeno-associated-virus-5	DNA-binding-trs-helicase	Tetraodon_nigroviridis.TETRAODON8.dna.nonchromosomal.fa	Un_random	94956190	94956660	471	99.4
Adeno-associated-virus-1	NS	336983_ref_Cang.pa_1.0_chrUn.fa	gi|786079843|ref|NW_012115044.1|	2545915	2546112	198	135
Adeno-associated-virus-1	VP	9531_ref_Caty_1.0_chrUn.fa	gi|778128673|ref|NW_012004166.1|	1691349	1691705	357	211
Adeno-associated-virus-1	VP	vicPac2.fa	KB632691	1513587	1513886	300	78.6
Adeno-associated-virus-1	VP	myoLuc2.fa	GL430312	465562	465975	414	122
Adeno-associated-virus-1	VP	myoLuc2.fa	GL430312	466775	467401	627	93.2
Adeno-associated-virus-1	VP	mbr_ref_ASM41265v1_chrUn.fa	gi|549497831|ref|NW_005366109.1|	2424667	2425335	669	114
Adeno-associated-virus-1	VP	mbr_ref_ASM41265v1_chrUn.fa	gi|549497831|ref|NW_005366109.1|	2423420	2423830	411	114
Adeno-associated-virus-1	VP	Myotis_dav_unplaced.scaf.fa_25.fa	gi|432086621|gb|KB116268.1|	748857	749219	363	184
Adeno-associated-virus-1	VP	macEug2.fa	GL067164	39183	39455	273	133
Adeno-associated-virus-1	VP	macEug2.fa	GL137465	2075	2239	165	63.5
Adeno-associated-virus-1	VP	macEug2.fa	GL111622	10412	10654	243	124
Adeno-associated-virus-2	NS	papAnu2.fa_3.fa	chr3	14387878	14389020	1143	754
Adeno-associated-virus-2	NS	papAnu2.fa_1.fa	chr11	48228919	48230019	1101	711
Adeno-associated-virus-2	NS	Chlorocebus_sabaeus.ChlSab1.1.dna.chromosome.1.fa	1_dna:chromosome_chromosome:ChlSab1.1:1:1:126035930:1_REF	79118998	79119483	486	293
Adeno-associated-virus-2	NS	9531_ref_Caty_1.0_chrUn.fa	gi|778117074|ref|NW_012006154.1|	2894332	2895426	1095	718
Adeno-associated-virus-2	NS	9531_ref_Caty_1.0_chrUn.fa	gi|778128673|ref|NW_012004166.1|	1692304	1693446	1143	735
Adeno-associated-virus-2	NS	C_lanigera_v0.assembly.fasta_5.fa	scaffold00012	22669255	22669584	330	103

Adeno-associated-virus-2	NS	C_lanigera_v0.assembly.fasta_5.fa	scaffold00012	22702389	22702709	321	95.9
Adeno-associated-virus-2	NS	hetGla2.fa_2.fa	JH602063	15521366	15521776	411	178
Adeno-associated-virus-2	NS	chr3.fa	chr3	12019929	12020633	705	105
Adeno-associated-virus-2	NS	criGri1.fa	KE377271	1016005	1016331	327	63.9
Adeno-associated-virus-2	NS	885580_ref_DMR_v1.0_chrUn.fa	gi|730053839|ref|NW_011044239.1|	3549538	3550038	501	75.9
Adeno-associated-virus-2	NS	885580_ref_DMR_v1.0_chrUn.fa	gi|730052864|ref|NW_011045214.1|	231618	232118	501	149
Adeno-associated-virus-2	NS	118797_ref_Lipotes_vexillifer_v1_chrUn.fa	gi|599273349|ref|NW_006777435.1|	1004199	1004375	177	63.9
Adeno-associated-virus-2	NS	74533_ref_PanTig1.0_chrUn.fa	gi|589289565|ref|NW_006711412.1|	1824011	1824391	381	77
Adeno-associated-virus-2	NS	AWGZ01.2.fsa_nt.fa	gi|539840960|gb|AWGZ01225077.1|	3964	4317	354	148
Adeno-associated-virus-2	NS	AWGZ01.3.fsa_nt.fa	gi|539768544|gb|AWGZ01297493.1|	8230	8433	204	85.9
Adeno-associated-virus-2	NS	myoLuc2.fa	GL430312	468449	468700	252	128
Adeno-associated-virus-2	NS	Eptesicus_f.unplaced.scaf.fa_17.fa	gi|411115880|gb|JH977683.1|	1718898	1719605	708	253
Adeno-associated-virus-2	NS	macEug2.fa	GL067164	38055	38744	690	318
Adeno-associated-virus-2	NS	macEug2.fa	GL092140	2	184	183	82.4
Adeno-associated-virus-2	VP	AGTM01.fasta_10.fa	gi|369230428|gb|AGTM011530899.1|	5870	5983	114	60.1
Adeno-associated-virus-2	VP	oryCun2.fa	chr10	8293766	8293984	219	73.9
Adeno-associated-virus-2	VP	turTru2.fa	JH472581	124620	124973	354	134
Adeno-associated-virus-2	VP	balAcu1.fa	KI538555	2065951	2066367	417	147
Adeno-associated-virus-2	VP	118797_ref_Lipotes_vexillifer_v1_chrUn.fa	gi|599249724|ref|NW_006783413.1|	1820807	1821175	369	103
Adeno-associated-virus-2	VP	ANOL02.fasta_13.fa	gi|442705876|gb|ANOL02028540.1|	35697	36050	354	137
Adeno-associated-virus-2	VP	AWHB01.2.fsa_nt.fa	gi|538698300|gb|AWHB01297376.1|	117	407	291	166
Adeno-associated-virus-2	VP	AWHB01.2.fsa_nt.fa	gi|538698301|gb|AWHB01297375.1|	760	1407	648	337
Adeno-associated-virus-2	VP	AWHB01.2.fsa_nt.fa	gi|538726583|gb|AWHB01269093.1|	30	251	222	79.3
Adeno-associated-virus-2	VP	AWHA01.3.fsa_nt.fa	gi|539420556|gb|AWHA01190251.1|	199	714	516	246
Adeno-associated-virus-2	VP	AWGZ01.3.fsa_nt.fa	gi|539768544|gb|AWGZ01297493.1|	10674	11009	336	135
Adeno-associated-virus-2	VP	AWGZ01.2.fsa_nt.fa	gi|539829657|gb|AWGZ01236380.1|	2708	3019	312	88.6
Adeno-associated-virus-2	VP	Eptesicus_f.unplaced.scaf.fa_17.fa	gi|411115880|gb|JH977683.1|	1720527	1721183	657	118
Adeno-associated-virus-2	VP	Eptesicus_f.unplaced.scaf.fa_17.fa	gi|411115880|gb|JH977683.1|	1722119	1722238	120	71.6
Adeno-associated-virus-2	VP	myoLuc2.fa	GL430312	467541	467870	330	113
Adeno-associated-virus-2	VP	proCap1.fa	scaffold_6934	37881	38063	183	87
Adeno-associated-virus-2	VP	macEug2.fa	GL067164	40844	41248	405	201
Adeno-associated-virus-2	VP	macEug2.fa	GL067164	39800	40246	447	216
Adeno-associated-virus-2	VP	macEug2.fa	GL155583	7170	7454	285	73.9
Adeno-associated-virus-3	NS	O_degus_v0.assembly.fasta_15.fa	scaffold00064	8427943	8429076	1134	528
Adeno-associated-virus-3	NS	AWHB01.2.fsa_nt.fa	gi|538698300|gb|AWHB01297376.1|	1060	1287	228	96.3
Adeno-associated-virus-3	VP	hetGla2.fa_3.fa	JH602093	17089028	17089267	240	81.6
Adeno-associated-virus-3	VP	hetGla2.fa_2.fa	JH602066	7342170	7342451	282	79
Adeno-associated-virus-3	VP	885580_ref_DMR_v1.0_chrUn.fa	gi|730053275|ref|NW_011044803.1|	11529215	11529409	195	85.1
Adeno-associated-virus-3	VP	118797_ref_Lipotes_vexillifer_v1_chrUn.fa	gi|599249724|ref|NW_006783413.1|	1821408	1821548	141	60.5
Adeno-associated-virus-3	VP	AWGZ01.2.fsa_nt.fa	gi|539840960|gb|AWGZ01225077.1|	1280	1666	387	102
Adeno-associated-virus-3	VP	Myotis_dav_unplaced.scaf.fa_25.fa	gi|432086621|gb|KB116268.1|	750202	750696	495	147
Adeno-associated-virus-3	VP	O_afer_v0.assembly.fa	scaffold00268	4836781	4837053	273	79
Adeno-associated-virus-3	VP	O_afer_v0.assembly.fa	scaffold00268	4837981	4838421	441	178
Adeno-associated-virus-4	NS	rn6.fa_3.fa	chr2	91608076	91608789	714	84.3
Adeno-associated-virus-4	NS	rn6.fa_3.fa	chr2	91676654	91677367	714	84.3
Adeno-associated-virus-4	NS	Mesocricetus_unplaced.scaf.fa_21.fa	gi|472278377|gb|KB708216.1|	1569487	1569924	438	82.8
Adeno-associated-virus-4	NS	chr8.fa	chr8	1277521	1277721	201	70.5
Adeno-associated-virus-4	NS	macEug2.fa	GL137465	650	1138	489	127
Adeno-associated-virus-5	DNA-binding-trs-helicase	oryCun2.fa	chr10	8294586	8295512	927	293
Adeno-associated-virus-5	DNA-binding-trs-helicase	ochPri3.fa_2.fa	JH802073	30948507	30949073	567	82.8
Adeno-associated-virus-5	DNA-binding-trs-	cerSim1.fa	JH767823	3032171	3032887	717	76.3

	helicase						
Adeno-associated-virus-5	DNA-binding-trs-helicase	AWGZ01.1.fsa_nt.fa	gil539959617lgblAWGZ01106480.1l	2213	2872	660	92.4
Adeno-associated-virus-5	DNA-binding-trs-helicase	Eptesicus_f.unplaced.scaf.fa_19.fa	gil411115864lgblJH977699.1l	1476841	1477455	615	83.2
Adeno-associated-virus-5	DNA-binding-trs-helicase	myoLuc2.fa	GL430312	469332	469580	249	80.5
Adeno-associated-virus-5	DNA-binding-trs-helicase	mbr_ref_ASM41265v1_chrUn.fa	gil549497831lreflNW_005366109.1l	2427256	2427507	252	86.3
Adeno-associated-virus-5	DNA-binding-trs-helicase	mbr_ref_ASM41265v1_chrUn.fa	gil549497831lreflNW_005366109.1l	2426376	2427080	705	233
Adeno-associated-virus-5	DNA-binding-trs-helicase	loxAfr3.fa	scaffold_4	61369624	61370745	1122	575
Adeno-associated-virus-5	DNA-binding-trs-helicase	sarHar1.fa_4.fa	chr3_GL849911_random	2300983	2301111	129	68.9
Adeno-associated-virus-5	VP	379532_ref_Pcoq_1.0_chrUn.fa	gil808384653lreflNW_012145967.1l	4720840	4721250	411	169
Adeno-associated-virus-5	VP	379532_ref_Pcoq_1.0_chrUn.fa	gil808385307lreflNW_012145313.1l	18594	19004	411	169
Adeno-associated-virus-5	VP	C_lanigera_v0.assembly.fasta_12.fa	scaffold00035	13652891	13653253	363	117
Adeno-associated-virus-5	VP	C_lanigera_v0.assembly.fasta_12.fa	scaffold00035	13653895	13654350	456	84.3
Adeno-associated-virus-5	VP	hetGla2.fa_3.fa	JH602093	17087381	17087614	234	92
Adeno-associated-virus-5	VP	hetGla2.fa_3.fa	JH602093	17088228	17088608	381	82
Adeno-associated-virus-5	VP	cavPor3.fa	scaffold_68	509024	509263	240	85.1
Adeno-associated-virus-5	VP	O_degus_v0.assembly.fasta_10.fa	scaffold00034	16377593	16377940	348	92.8
Adeno-associated-virus-5	VP	chr3.fa	chr3	12016997	12017200	204	92.8
Adeno-associated-virus-5	VP	885580_ref_DMR_v1.0_chrUn.fa	gil730053275lreflNW_011044803.1l	11530014	11530247	234	82.4
Adeno-associated-virus-5	VP	885580_ref_DMR_v1.0_chrUn.fa	gil730053275lreflNW_011044803.1l	11530831	11531157	327	105
Adeno-associated-virus-5	VP	oryCun2.fa	chr10	8292880	8293209	330	110
Adeno-associated-virus-5	VP	oryCun2.fa	chr10	8292091	8292387	297	77.8
Adeno-associated-virus-5	VP	118797_ref_Lipotes_vexillifer_v1_chrUn.fa	gil599249724lreflNW_006783413.1l	1822634	1822987	354	136
Adeno-associated-virus-5	VP	cerSim1.fa	JH767727	28719851	28720156	306	83.6
Adeno-associated-virus-5	VP	canFam3.fa	chr37	9272234	9272599	366	155
Adeno-associated-virus-5	VP	AWHA01.3.fsa_nt.fa	gil539377818lgblAWHA01212176.1l	1715	1987	273	134
Adeno-associated-virus-5	VP	AWHA01.3.fsa_nt.fa	gil539420557lgblAWHA01190250.1l	3674	3874	201	97.8
Adeno-associated-virus-5	VP	AWGZ01.2.fsa_nt.fa	gil539840960lgblAWGZ01225077.1l	2077	3168	1092	363
Adeno-associated-virus-5	VP	Eptesicus_f.unplaced.scaf.fa_17.fa	gil411115880lgblJH977683.1l	1720172	1720369	198	95.5
Adeno-associated-virus-5	VP	Myotis_dav_unplaced.scaf.fa_25.fa	gil432086621lgblKB116268.1l	748398	748598	201	92
Adeno-associated-virus-5	VP	mbr_ref_ASM41265v1_chrUn.fa	gil549497831lreflNW_005366109.1l	2425591	2425782	192	84.3
Adeno-associated-virus-5	VP	sarHar1.fa_3.fa	chr3_GL849578_random	682378	682512	135	62.4
Adeno-associated-virus-7	NS	hetGla2.fa_4.fa	JH602103	4913667	4914293	627	103
Adeno-associated-virus-7	NS	AWHA01.3.fsa_nt.fa	gil539420557lgblAWHA01190250.1l	1859	2995	1137	314
Adeno-associated-virus-7	NS	Myotis_dav_unplaced.scaf.fa_25.fa	gil432086621lgblKB116268.1l	747054	747551	498	226
Adeno-associated-virus-7	VP	AWGZ01.3.fsa_nt.fa	gil539768544lgblAWGZ01297493.1l	9155	9466	312	119
Adeno-associated-virus-7	VP	dasNov3.fa	JH575770	698735	699205	471	250
Adeno-associated-virus-8	NS	tarSyr1.fa_5.fa	scaffold_178296	1024	1968	945	82.8
Adeno-associated-virus-8	VP	papAnu2.fa_3.fa	chr3	14389616	14389918	303	159
Adeno-associated-virus-8	VP	AGTM01.fasta_34.fa	gil368868552lgblAGTM011892775.1l	752	1168	417	161
Adeno-associated-virus-8	VP	AGTM01.fasta_8.fa	gil369241804lgblAGTM011519523.1l	856	1272	417	111
Adeno-associated-virus-8	VP	cerSim1.fa	JH767777	15368624	15369037	414	168
Adeno-associated-virus-8	VP	AWHA01.3.fsa_nt.fa	gil539377818lgblAWHA01212176.1l	3313	3729	417	211
Adeno-associated-virus-8	VP	AWGZ01.4.fsa_nt.fa	gil539662995lgblAWGZ01402898.1l	9078	9506	429	131
Adeno-associated-virus-8	VP	proCap1.fa	scaffold_6934	39408	39620	213	107
Avian-adeno-associated-virus-ATCC-VR-865	NS	chrX.fa	gil441468466lgblCM001739.1l	4735360	4735524	165	63.2
Avian-adeno-associated-virus-ATCC-	NS	oviAri3.fa	chrX	129362133	129362297	165	65.9

VR-865							
Avian-adeno-associated-virus-ATCC-VR-865	NS	bosTau7.fa	chrUn_JH121315	733718	733882	165	63.2
Avian-adeno-associated-virus-ATCC-VR-865	NS	bbu_ref_UMD_CASPUR_WB_2.0_chrUn.fa	gil551721726lreflNW_005784554.1l	486077	486241	165	63.2
Avian-adeno-associated-virus-ATCC-VR-865	NS	unplaced.scaf.fa_6.fa	gil431914203lgblKB030536.1l	7950788	7951021	234	62.4
Avian-adeno-associated-virus-ATCC-VR-865	NS	pteVam1.fa	scaffold_33	547207	547440	234	60.1
Avian-adeno-associated-virus-ATCC-VR-865	VP	hetGla2.fa_2.fa	JH602066	7341412	7341999	588	72
Avian-adeno-associated-virus-strain-DA-1	NS	turTru2.fa	JH472581	127388	128143	756	120
Bat-adeno-associated-virus-YNM	NS	oro_ref_Oros_1.0_chrUn.fa_33.fa	gil469043811lreflNW_004451877.1l	138390	138698	309	69.3
Bat-adeno-associated-virus-YNM	NS	O_afer_v0.assembly.fa	scaffold00268	4835564	4835764	201	81.3
Bat-adeno-associated-virus-YNM	VP	chr3.fa	chr3	12017830	12018192	363	60.8
Bovine-adeno-associated-virus	NS	C_lanigera_v0.assembly.fasta_15.fa	scaffold00050	18094567	18095718	1152	455
Bovine-adeno-associated-virus	NS	cavPor3.fa	scaffold_28	20603427	20603621	195	80.9
Bovine-adeno-associated-virus	NS	cavPor3.fa	scaffold_35	10624000	10624824	825	138
Bovine-adeno-associated-virus	NS	rn6.fa_3.fa	chr19	15282969	15283322	354	61.2
Bovine-adeno-associated-virus	NS	Myotis_dav_unplaced.scaf.fa_25.fa	gil432086621lgblKB116268.1l	746455	746706	252	81.6
Bovine-adeno-associated-virus	NS	proCap1.fa	scaffold_19252	22396	23172	777	188
Bovine-adeno-associated-virus	NS	macEug2.fa	GL137465	1290	1481	192	92.4
Bovine-adeno-associated-virus	VP	macEug2.fa	GL115959	11007	11423	417	177
Goose-parvovirus	NS	chr8.fa	chr8	93163708	93163959	252	65.5
Goose-parvovirus	NS	balAcu1.fa	KI538555	2062632	2063012	381	107
Goose-parvovirus	NS	ANOL02.fasta_13.fa	gil442705876lgblANOL02028540.1l	32498	33043	546	151
Goose-parvovirus	NS	cerSim1.fa	JH767777	15365258	15365440	183	72
Goose-parvovirus	NS	74533_ref_PanTig1.0_chrUn.fa	gil589288889lreflNW_006712088.1l	732703	732855	153	64.7
Goose-parvovirus	NS	unplaced.scaf.fa_3.fa	gil472269955lgblKB714734.1l	2752124	2752417	294	63.5
Goose-parvovirus	NS	AWHA01.3.fsa_nt.fa	gil539429327lgblAWHA01181480.1l	14356	14577	222	66.6
Muscovy-duck-parvovirus	NS	AGTM01.fasta_10.fa	gil369230428lgblAGTM011530899.1l	3995	4885	891	241
Muscovy-duck-parvovirus	NS	turTru2.fa	JH472581	128469	128720	252	90.9
Muscovy-duck-parvovirus	NS	118797_ref_Lipotes_vexillifer_v1_chrUn.fa	gil599249724lreflNW_006783413.1l	1818823	1819074	252	99.8
Muscovy-duck-parvovirus	NS	ANOL02.fasta_13.fa	gil442705876lgblANOL02028540.1l	31923	32219	297	94.4
Muscovy-duck-parvovirus	NS	cerSim1.fa	JH767777	15365655	15366053	399	159
Muscovy-duck-parvovirus	NS	unplaced.scaf.fa_10.fa	gil431908567lgblKB030662.1l	2359689	2359865	177	85.9
Muscovy-duck-parvovirus	NS	pteVam1.fa	scaffold_4823	93847	94014	168	89.7
Muscovy-duck-parvovirus	NS	GCA_000465285.1_ASM46528v1_genomic.fa	KE761265.1	27592	27810	219	90.5
Muscovy-duck-parvovirus	NS	AWGZ01.3.fsa_nt.fa	gil539768544lgblAWGZ01297493.1l	7317	7784	468	142
Muscovy-duck-parvovirus	NS	macEug2.fa	GL092140	4803	5018	216	80.1
Muscovy-duck-parvovirus	NS	macEug2.fa	GL077261	32888	33547	660	120
Muscovy-duck-parvovirus	NS	sarHar1.fa_6.fa	chr5_GL861700_random	1041966	1042724	759	90.5
Muscovy-duck-parvovirus	NS	ornAna1.fa	chr2	47023240	47023464	225	71.2
Muscovy-duck-parvovirus	NS	ornAna1.fa	Contig18075	18760	19056	297	74.7
Muscovy-duck-parvovirus	VP	turTru2.fa	JH472581	126465	126665	201	75.9
Muscovy-duck-parvovirus	VP	ANOL02.fasta_13.fa	gil442705876lgblANOL02028540.1l	33974	34165	192	75.1
Muscovy-duck-parvovirus	VP	Camelus_f.unplaced.scaf.fa_14.fa	gil429342667lgblKB017125.1l	3396540	3396749	210	75.1
Muscovy-duck-parvovirus	VP	cerSim1.fa	JH767777	15367090	15367350	261	83.2
Snake-adeno-associated-virus	VP	AGTM01.fasta_4.fa	gil370174340lgblAGTM010595279.1l	156	401	246	113
Snake-adeno-associated-virus	VP	AWGZ01.2.fsa_nt.fa	gil539829657lgblAWGZ01236380.1l	1773	1979	207	77.4
Adeno-associated-virus-2	NS	146911_ref_Gekko_japonicus_V1.1_chrUn.fa	gil972885930lreflNW_015177013.1l	278010	278207	198	71.2
Bovine-parvovirus	hypothetical-protein	Latimeria_chalumnae.LatCha1.dna.toplevel.fa	JH127032.1	1275425	1276297	873	124

Bovine-parvovirus	hypothetical-protein	AWGZ01.2.fsa_nt.fa	gi|539829657|gb|AWGZ01236380.1|	1233	1439	207	84.7
Bovine-parvovirus-2	NS	Myotis_dav_unplaced.scaf.fa_11.fa	gi|432104950|gb|KB106247.1|	543	908	366	70.5
Canine-parvovirus	NS	macEug2.fa	GL142423	26995	27204	210	63.2
Canine-parvovirus	NS	macEug2.fa	GL109622	44978	45490	513	85.9
Canine-parvovirus	VP	C_lanigera_v0.assembly.fasta_24.fa	scaffold00132	459313	459717	405	89.4
Canine-parvovirus	VP	cavPor3.fa	scaffold_27	12842929	12843246	318	76.6
Canine-parvovirus	VP	rn6.fa_4.fa	chr5	68317391	68318704	1314	576
Canine-parvovirus	VP	triMan1.fa_2.fa	JH594625	3922931	3923245	315	96.3
Canine-parvovirus	VP	triMan1.fa_2.fa	JH594625	3922000	3922350	351	91.7
Canine-parvovirus	VP	macEug2.fa	GL146523	7340	7921	582	60.8
Goose-parvovirus	VP	AGTM01.fasta_8.fa	gi|369241804|gb|AGTM011519523.1|	1	423	423	177
Goose-parvovirus	VP	turTru2.fa	JH472581	125606	126163	558	238
Goose-parvovirus	VP	AWHB01.3.fsa_nt.fa	gi|538561164|gb|AWHB01434492.1|	2	397	396	89.7
Goose-parvovirus	VP	AWGZ01.3.fsa_nt.fa	gi|539768544|gb|AWGZ01297493.1|	9544	10245	702	245
Goose-parvovirus	VP	macEug2.fa	GL155583	8579	8836	258	90.9
Minute-virus-of-mice	hypothetical-protein-MMVgp3	rn6.fa_4.fa	chr5	68319258	68319566	309	171
Minute-virus-of-mice	hypothetical-protein-MMVgp3	O_afer_v0.assembly.fa	scaffold00073	9405422	9405664	243	87
Minute-virus-of-mice	hypothetical-protein-MMVgp3	O_afer_v0.assembly.fa	scaffold00132	915464	915727	264	73.9
Minute-virus-of-mice	hypothetical-protein-MMVgp3	chr6.fa	chr6	48170130	48170384	255	68.6
Minute-virus-of-mice	hypothetical-protein-MMVgp3	macEug2.fa	GL161406	13249	13512	264	93.2
Minute-virus-of-mice	hypothetical-protein-MMVgp3	macEug2.fa	GL152084	12707	12958	252	106
Minute-virus-of-mice	hypothetical-protein-MMVgp3	macEug2.fa	GL096756	10393	10692	300	83.6
Minute-virus-of-mice	hypothetical-protein-MMVgp3	macEug2.fa	GL132395	8167	8589	423	84.7
Minute-virus-of-mice	hypothetical-protein-MMVgp3	sarHar1.fa_1.fa	chr1_GL834466_random	3164454	3164651	198	67
Minute-virus-of-mice	NS	O_degus_v0.assembly.fasta_21.fa	scaffold00109	3857839	3858054	216	100
Minute-virus-of-mice	NS	rn6.fa_4.fa	chr5	68316575	68316787	213	130
Minute-virus-of-mice	NS	C_asiatica_v0.assembly.fa	scaffold00156	3606438	3606581	144	77.4
Minute-virus-of-mice	NS	sarHar1.fa_3.fa	chr2_GL842354_random	121536	121976	441	92.8
Minute-virus-of-mice	NS	sarHar1.fa_4.fa	chr3_GL849626_random	4354498	4354794	297	60.8
Minute-virus-of-mice	NS	sarHar1.fa_6.fa	chr6_GL864827_random	1912708	1912893	186	85.9
Porcine-parvovirus	VP	C_lanigera_v0.assembly.fasta_12.fa	scaffold00033	14638784	14639092	309	108
Porcine-parvovirus	VP	C_lanigera_v0.assembly.fasta_24.fa	scaffold00132	461275	461496	222	87.4
Porcine-parvovirus	VP	C_lanigera_v0.assembly.fasta_12.fa	scaffold00033	14636960	14637289	330	80.9
Porcine-parvovirus	VP	O_degus_v0.assembly.fasta_33.fa	scaffold00312	1343571	1343834	264	95.1
Porcine-parvovirus	VP	O_degus_v0.assembly.fasta_21.fa	scaffold00109	3857142	3857345	204	85.1
Porcine-parvovirus	VP	O_degus_v0.assembly.fasta_18.fa	scaffold00088	368258	368782	525	134
Porcine-parvovirus	VP	C_asiatica_v0.assembly.fa	scaffold00010	34331390	34331743	354	75.5
Porcine-parvovirus	VP	chr3.fa	chr3	352564966	352565232	267	96.7
Porcine-parvovirus	VP	chr6.fa	chr6	113563927	113564157	231	91.3
Porcine-parvovirus	VP	chr6.fa	chr6	48169075	48169314	240	111
Porcine-parvovirus	VP	chr8.fa	chr8	230394727	230394984	258	89
Porcine-parvovirus	VP	macEug2.fa	GL084187	8943	9176	234	84.3
Porcine-parvovirus	VP	macEug2.fa	GL150710	17277	17588	312	106
Porcine-parvovirus	VP	macEug2.fa	GL081802	59178	59423	246	98.6

Porcine-parvovirus	VP	sarHar1.fa_1.fa	chr1_GL834608_random	1658482	1658721	240	72
Porcine-parvovirus-6	NS	AWHA01.2.fsa_nt.fa	gi|539521749|gb|AWHA01101022.1|	15939	16418	480	103
Porcine-parvovirus-6	NS	unplaced.scaf.fa_5.fa	gi|431914431|gb|KB030533.1|	3953770	3954243	474	103
Porcine-parvovirus-6	NS	pteVam1.fa	scaffold_5601	29365	29841	477	89.4

*NCBI reference virus protein sequences, downloaded March 2016

Table S3. Overlaps between NS and VP genes in the subfamily *Parvovirinae*.

	GenBank	Virus	Strain	Overlap
Chapparvoviruses	Our Study	Desmodus rotundus parvovirus	-	8 nucleotides
	Our Study	Cebus capucinus imitator parvovirus	-	8 nucleotides
	Our Study	Mesitornis unicolor isolate parvovirus	-	8 nucleotides
	Our Study	Protobothrops mucrosquamatus parvovirus	-	11 nucleotides
	KF925531	Turkey parvovirus	TP1-2012/HUN	8 nucleotides
	KU563733	Porcine parvovirus 7	42	No
	KX272741	Rat parvovirus 2	9	No
Erythroparvovirus	M13178	human parvovirus B19	Au	8 nucleotides
	U26342	Simian parvovirus	-	8 nucleotides
	AF221123	Pig-tailed macaque parvovirus	-	11 nucleotides
	GQ200736	Chipmunk parvovirus	-	None
	AF221123	Bovine parvovirus 3	-	None
